# High taxonomic resolution surveys and trait-based analyses reveal multiple benthic regimes in North Sulawesi (Indonesia)

**DOI:** 10.1038/s41598-021-95905-8

**Published:** 2021-08-16

**Authors:** Miriam Reverter, Matthew Jackson, Sven Rohde, Mareen Moeller, Robert Bara, Markus T. Lasut, Marco Segre Reinach, Peter J. Schupp

**Affiliations:** 1 Institute for Chemistry and Biology of the Marine Environment (ICBM) at the Carl Von Ossietzky University of Oldenburg, Wilhelmshaven, Germany; 2 Faculty of Fisheries and Marine Science, Sam Ratulangi University, Jl. Kampus UNSRAT Bahu, 95115 Manado, Sulawesi Utara Indonesia; 3 Coral Eye, Bangka Island, North Sulawesi Indonesia; 4 Helmholtz Institute for Functional Marine Biodiversity at the University of Oldenburg (HIFMB), 26129 Oldenburg, Germany

**Keywords:** Biodiversity, Community ecology

## Abstract

As coral reef communities change and reorganise in response to increasing disturbances, there is a growing need for understanding species regimes and their contribution to ecosystem processes. Using a case study on coral reefs at the epicentre of tropical marine biodiversity (North Sulawesi, Indonesia), we explored how application of different biodiversity approaches (i.e., use of major taxonomic categories, high taxonomic resolution categories and trait-based approaches) affects the detection of distinct fish and benthic communities. Our results show that using major categories fails to identify distinct coral reef regimes. We also show that monitoring of only scleractinian coral communities is insufficient to detect different benthic regimes, especially communities dominated by non-coral organisms, and that all types of benthic organisms need to be considered. We have implemented the use of a trait-based approach to study the functional diversity of whole coral reef benthic assemblages, which allowed us to detect five different community regimes, only one of which was dominated by scleractinian corals. Furthermore, by the parallel study of benthic and fish communities we provide new insights into key processes and functions that might dominate or be compromised in the different community regimes.

## Introduction

Ecosystems worldwide are experiencing profound ecological changes including biodiversity losses^1^ and community rearrangements (i.e., non-random species turnover)^2^, which are expected to worsen with climate change, even under moderate CO_2_ mitigation scenarios^3^. Non-random species turnover, which depends on the susceptibility of the organisms’ traits, can disrupt vital ecosystem processes such as trophic energy flow^4^ or habitat provisioning^5^, deeply affecting ecosystem functioning and resilience^6^. Understanding distinct and emerging species configurations and their contribution to key ecosystem functions is therefore needed to establish effective conservation and management strategies^7,8^.

Over the past four decades, tropical coral reefs, one of Earth’s most biodiverse ecosystems, have experienced global declines and shifts in species compositions that deeply affect their functioning and the ecosystem services provided^2,9,10^. A turnover from highly three-dimensional scleractinian corals such as Acroporidae to more robust corals (e.g., Poritidae), has been observed worldwide after acute disturbances, such as bleaching events or crown-of-thorns outbreaks^2,11^. Shifts in species compositions including decreases in scleractinians and increases in non-reef building species such as algae, sponges and octocorals are also becoming more frequent as a result of continuous anthropogenic and climate stressors^12–14^. Such compositional changes affect several core ecosystem processes (i.e., carbonate production, primary production, trophic interactions and reef replenishment) and pose new conservation challenges^5,15,16^.

Coral reefs are heterogeneous ecosystems, with highly varied biological communities that depend on both the local physical environment (e.g., reef topography, wave exposure) and larger biogeographic patterns^17,18^. Compositionally and functionally distinct ecosystems will likely respond differently to disturbances, which can then result in different species configurations, further hindering the study and prediction of coral reef trajectories and their effect on core ecosystem processes^17,19^. In this context, conservation approaches need to consider both coral reefs spatio-temporal heterogeneity (i.e., different species configurations) and their contribution towards core ecosystem processes^20^.

The study of species configurations (i.e., community biodiversity) has been traditionally studied as the relative abundance of different taxa, and as such, most studies assessing coral reef composition have mostly used taxonomic categories (often at family level or higher, especially for benthic organisms) to identify community changes^21,22^. Approaches using major taxonomic categories (e.g., hard coral, soft coral, algae, etc.), have the advantage of being easily implemented in global citizen science programs and have allowed identification of marked regime shifts, for example, from coral to algae-dominated communities^22–24^. However, the use of major benthic categories might overlook functionally important compositional changes^25^. Many studies have shown that different species contribute differently to ecosystem functioning, and therefore ecological research has seen a shift from taxonomic diversity to functional diversity studies^26,27^. In functional diversity analyses, organisms are classified according to their life traits or functions, which allows identifying community-level changes in mean community traits. In fact, the information provided by the functional structure of communities is nowadays considered as a key indicator of the ecological status and resilience of an ecosystem^28^. Trait-based approaches therefore offer new opportunities for a deeper mechanistic understanding on the role of biodiversity in maintaining multiple ecosystem processes and they allow identification of species with critical and vulnerable ecosystem functions^29,30^. Trait-based approaches have been successfully used to study changes in coral reef fish communities^29,31^ and in scleractinian coral assemblages^26,32,33^. However, whilst scleractinian corals are the key organisms of coral reefs, recent shifts towards assemblages dominated by alternate organisms highlight the need of expanding these approaches to include all types of benthic organisms.

Here, we studied coral reefs around Bangka and Bunaken islands (North Sulawesi, Indonesia), which are at the epicentre of marine biodiversity^34^ and display high spatial heterogeneity^17,35^. Site characteristics such as wave exposure, depth and local anthropogenic stressors such as pollution or fishing are strong determinants of communities’ compositions^36–38^. For example, in coral reefs, overfishing (loss of top down control) and eutrophication (loss of bottom up control) have been recurrently associated to coral-algal regimes shifts. Sedimentation and turbidity have also been observed to drive shifts towards regimes dominated by algae, sponges, or zoanthids^39–41^. Here, we studied reefs with different topographies and exposed to different anthropogenic pressures as a case study to explore how the use of different biodiversity approaches (major taxonomic categories, high taxonomic resolution categories and trait-based approaches) affects the detection of distinct community (benthic and fish) compositions. We also implemented the use of a trait-based approach to study the functional diversity of coral reef benthic assemblages, including all types of sessile organisms encountered (e.g., sponges, ascidians, soft corals, etc.). Furthermore, we also analysed how the determination of distinct benthic regimes (defined using a cluster analyses) change when commonly used groups such as scleractinian corals or reef fish are complemented with all sessile benthic organisms.

## Results

### Identifying community patterns

**Figure 1:**
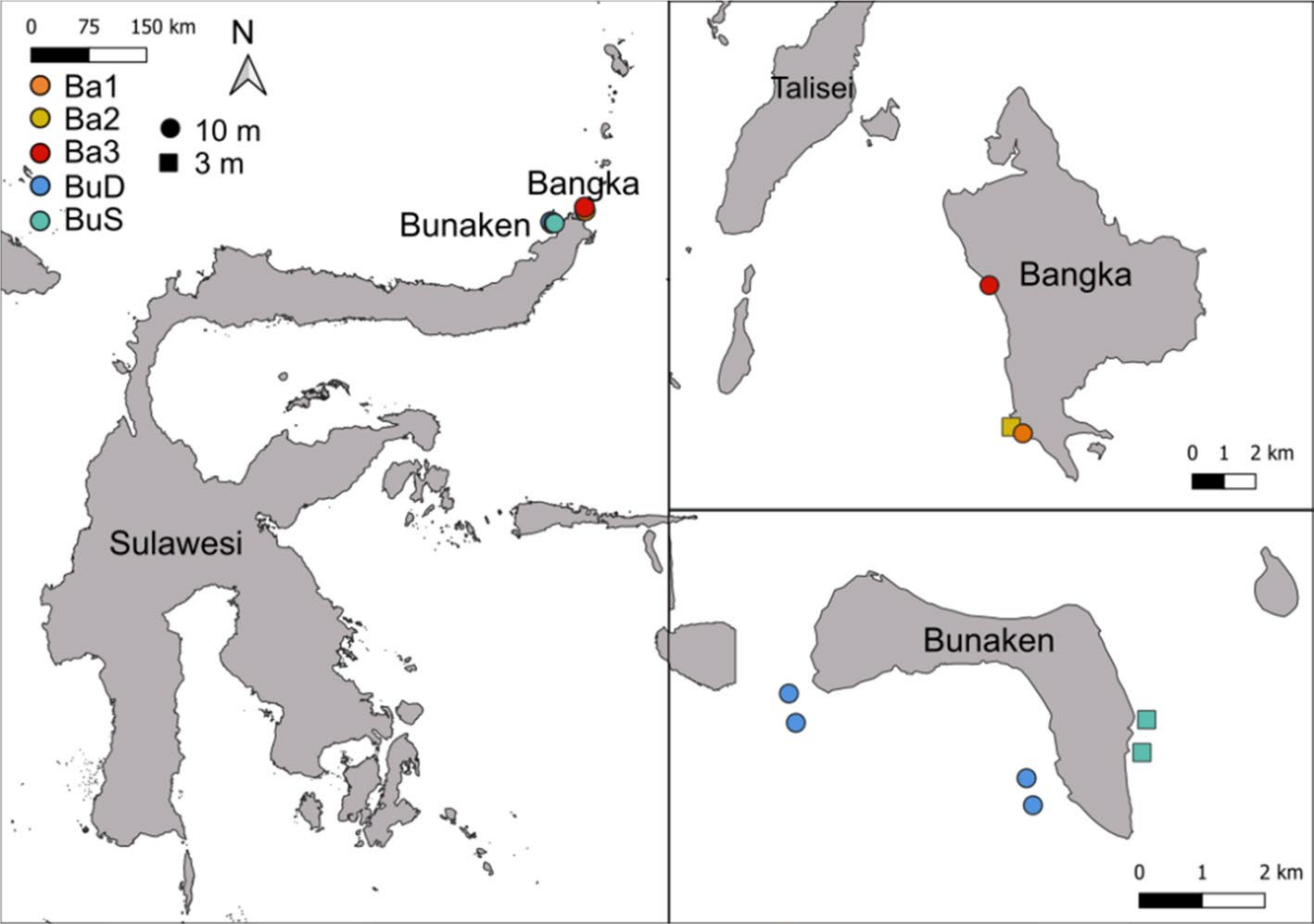
Map of the sites monitored around Bangka and Bunaken Islands (North Sulawesi, Indonesia). Colours indicate the different regimes identified. This map was created using QGIS software (QGIS.org, 2021. QGIS Geographic Information System. QGIS Association. http://www.qgis.org).

**Figure 2:**
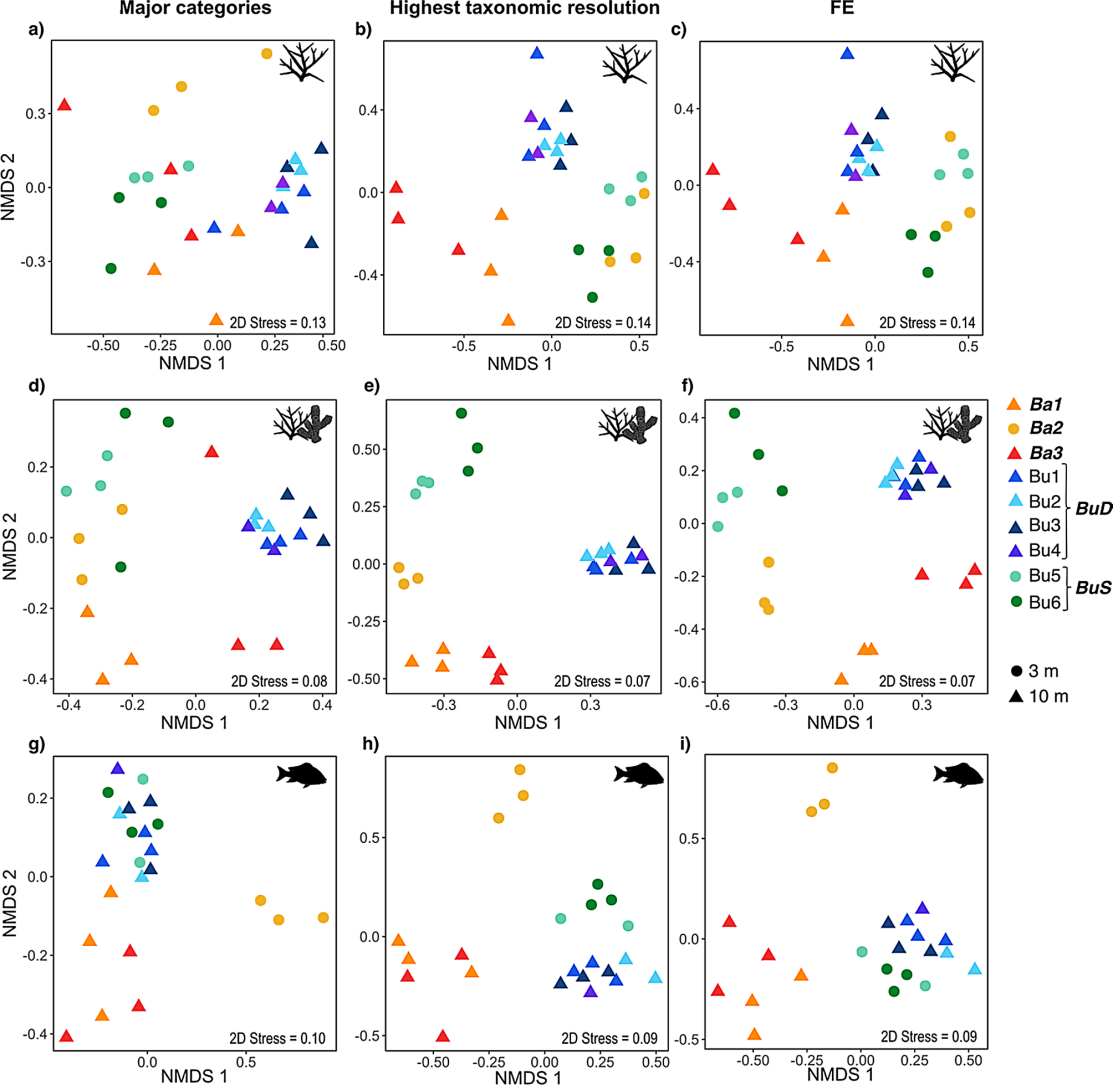
Analysis of the benthos [only scleractinian corals (**a**–**c**) and all benthic organisms (**d**–**f**)] and fish (**g**–**i**) community similarities (nMDS based on Euclidean distances) between the different sites samples using three levels of ecological information: major categories, highest taxonomic resolution and functional entities (FE). Community regimes of sites grouping together are highlighted in bold and italics (Ba1, Ba2, Ba3, BuD and BuS).

We studied the benthic (scleractinian corals and all benthic organisms) and fish community composition of nine coral reefs in North Sulawesi (Indonesia, Supplementary Table 1) by using major categories, categories at the highest taxonomic resolution possible and functional entities (FEs, defined using trait-base approaches) (Supplementary Tables 2, 3). The analysis of high taxonomic data and FEs showed marked differences between the benthos (all benthic organisms) and fish communities of the sites studied, allowing the identification of five significantly different regimes (i.e., sites with similar compositions identified using a cluster analysis) (Figs. 1, 2, Supplementary Figure 1). We identified three distinct regimes in Bangka, corresponding to the three sites studied (Ba1, Ba2 and Ba3) and two regimes in Bunaken, which grouped the deep sites (BuD regime containing Bu1, Bu2, Bu3 and Bu4) and the shallow sites (BuS regime, containing Bu5 and Bu6) (Figs. 1, 2, Supplementary Figure 1).

Regardless of the type of community studied (i.e., only scleractinian corals, all benthic organisms, or fish) the use of major categories failed to identify most of the community differences highlighted by the use of higher resolution data (i.e., high taxonomic resolution or FEs) (Fig. 2). Whereas it was to be expected that major categories would result in a lower regime identification (i.e., fewer input variables therefore fewer resulting clusters), here we show that the identified regimes using major categories are less consistent through the different datasets (corals, benthos, fish). For example, the analysis of coral major categories (i.e., classified upon their morphology), only allowed the clear identification of BuD regime, whereas all the other Bunaken and Bangka sites were distributed into three mixed clusters (Fig. 2a, Supplementary Figure 1). Similarly, the analysis of the coral reef benthos using major categories resulted in the identification of four clusters, two of which contained transects from BuD and Ba3 respectively, whereas the two remaining mixed transects from Ba2, Ba1 and BuS. Finally, the fish family analysis only allowed the identification of a significantly different regime in Ba2, which was not identified in the other datasets and grouped together the other Bangka (Ba1 and Ba3) and Bunaken sites into two other clusters (Figs. 1, 2 Supplementary Figure 1).

The results also show that the study of all benthic organisms allows much better detection of different community regimes than just the study of scleractinian coral communities. For example, the sites from Ba2 grouped together with the shallow Bunaken sites (BuS) when using only scleractinian corals, but were clearly distinguished when all the benthic organisms were considered (Fig. 2, Supplementary Figure 1).

### Benthic community structure

**Figure 3:**
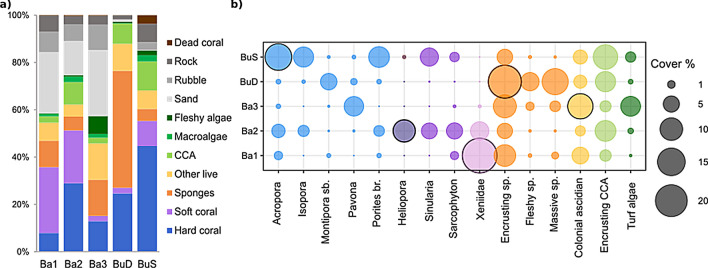
Benthic composition (based on major categories) of the different community regimes identified (**a**) and cover (%) of the most abundant (≥ 5% at least in one regime) benthic taxonomic categories identified (**b**). Highlighted circles represent the dominant benthic organism in each of the communities.

Our dataset was composed of highly heterogeneous reefs, with each of the regimes detected using high resolution taxonomic data and trait-based analyses dominated by different benthic organisms: scleractinian hard corals (BuS), blue coral *Heliopora coerulea* (Ba2), xenid soft corals (Ba1), colonial ascidians (Ba3) and sponges (BuD) (Fig. 3a,b).

Only two (Ba2 and BuS) out of the five regimes identified were dominated by reef-building species (i.e., hard corals, 28.9 ± 6.5% and 44.7 ± 15.6%, respectively). The reef at Ba2 was predominantly dominated by the blue coral *H. coerulea* (9.5 ± 3.7%), with lower covers of branching scleractinian corals (*Acropora* spp. 3.4 ± 3.5% and *Porites* spp. 2.1 ± 0.6%) and columnar coral *Isopora palifera* (2.4 ± 2.4%) (Fig. 3a,b). Ba2 also displayed important covers of encrusting coralline algae (8.5 ± 0.3%) and soft corals from the Alcyoniidae (mainly *Sarcophyton* spp. 5.0 ± 3.4% and *Sinularia* spp. 4.8 ± 2.1%) and Xeniidae (6.6 ± 2.3%) families. BuS reefs were dominated by branching scleractinian corals (*Acropora* spp. 13.7 ± 16.9% and *Porites* spp. 8.1 ± 6%), the columnar coral *Isopora palifera* (7.8 ± 5.9%) and massive *Porites* spp. (4.4 ± 4.0%). BuS sites also displayed large covers of encrusting coralline algae (11.7 ± 2.6%) and Alcyoniidae corals (mostly *Sinularia* spp. 6.3 ± 5.9%) (Fig. 3a,b).

Ba1 transects were dominated by soft corals from the Xeniidae family (23.5 ± 3.0%), followed by incrusting sponges (9.2 ± 3.6%), and presented low hard coral cover (7.9 ± 1.3%). Ba3 was dominated by sponges (15.2 ± 3.1%), mostly encrusting sponges (10.3 ± 1.3%), followed by ascidians (13.4 ± 4.4%, mostly encrusting colonial ascidians) and hard corals (12.9 ± 8.1%, mostly *Pavona* spp.). Sponges accounted for 49.4 ± 6.5% of the cover in BuD transects, with encrusting sponges being the most abundant organisms (21.2 ± 4.2%), followed by massive (13.4 ± 3.8%) and fleshy encrusting sponges (6.4 ± 1.5%). Hard coral cover was 24.7 ± 4.9%, with submassive *Montipora* ssp. (4.2 ± 2.9) and massive *Porites* spp. (3.1 ± 1.4%) being the most abundant genus (Fig. 3a,b).

### Functional diversity analysis of benthos communities

**Figure 4:**
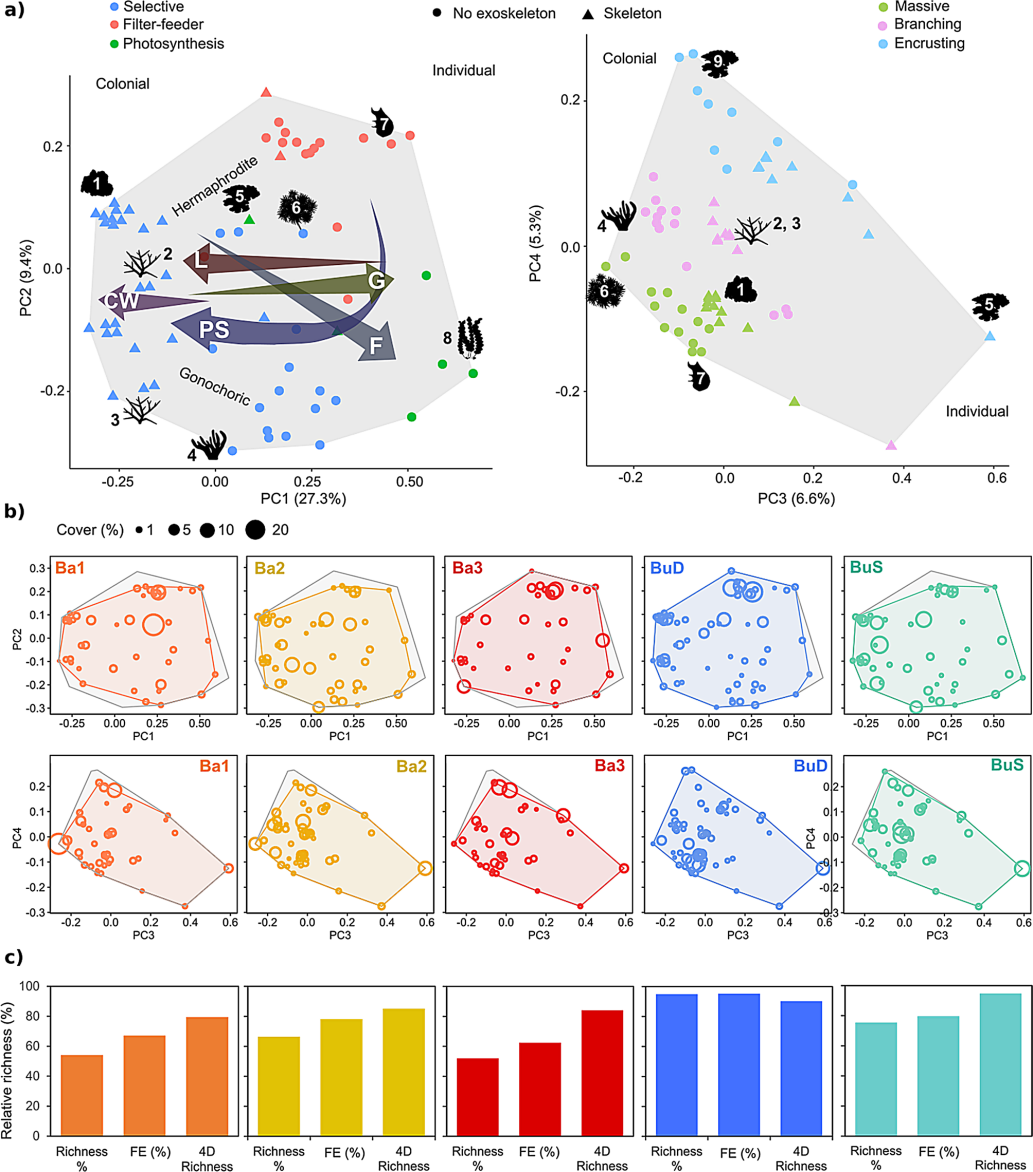
Benthic functional diversity of the different communities identified. (**a**) Distribution of functional entities (FEs) in the global benthic functional space, built using four PCoA axis (PC1 and PC2 left, PC3 and PC4 right) using twelve functional traits: colony formation, growth form, maximum colony size, longevity (L), growth rate (G), body flexibility (F), skeleton presence, reproductive strategy, sexual system, feeding strategy, presence of photosynthetic symbionts (PS) and corallite maximum width (CW, only for scleractinian corals). The numbers indicate the following functional entities: 1: massive hermaphrodite scleractinian corals, 2: branching hermaphrodite scleractinian corals, 3: branching gonochoric scleractinian corals, 4: *Sinularia* soft coral, 5: encrusting crustose coralline algae, 6: Xeniidae soft coral, 7: solitary ascidians, 8: macroalgae, 9: encrusting filter-feeders (sponges and ascidians). (**b**) Functional spaces of each of the communities analysed (coloured convex hull) superposed to the global functional space. (**c**) Functional diversity indices for each of the communities: relative taxonomic richness (Richness %), relative FE richness (FE %) and relative functional richness as % of filled global functional space (4D richness).

We identified 99 high taxonomic resolution benthic categories that were classified into 64 FEs (Supplementary Table 2) for which their functional niche was displayed using a functional space built on four PCoA axis. Generally, species longevity, corallite maximum width (for scleractinian corals), flexibility and growth rate changed along the first axis (PC1) (Fig. 4a). Colony form was highly structured along the fourth axis (PC4) (Fig. 4a). 29 out of the 64 FEs contained calcified species contributing to reef accretion (e.g., hard corals, crustose coralline algae, foraminifera), with nine FEs also contributing to reef structural complexity (i.e., branching morphology). 25 FEs out of the 64 FEs identified contained fast-growing species, including some potentially proliferating species (e.g., cyanobacteria, macroalgae, encrusting sponges, encrusting ascidians).

The three Bangka sites displayed the lowest taxonomic and FE richness (S_richness_ = 52–67%, FE_richness_ = 63–78%), but still filled 80 (Ba1), 85 (Ba2) and 84% (Ba3) of the benthos functional space (Fig. 4b,c). Only Ba2 was characterised (e.g., community-weighted means of trait values, CWM) by reef-building species (branching long-lived calcified species with fast growth) (Table 1, Fig. 4b). Ba2 contained 24 out of the 29 FEs contributing to reef accretion and the 9 FEs also contributing to reef structural complexity (Fig. 4b). In contrast, Ba1 and Ba3 were characterised by fast-growing short-lived species with no contribution to reef accretion (i.e., no skeleton) (Table 1, Fig. 4b). Ba1 and Ba3 also displayed the lowest functional diversity of reef-building species, with 19 and 18 FEs contributing to reef accretion, respectively. Ba1 contained only five FEs contributing simultaneously to reef accretion and reef structural complexity, but the cover of these FEs was extremely low (< 3%). Ba3 contained only four branching reef-building FEs, with the FE containing *Pavona* spp. reaching 7% of the benthic cover. Ba3 also displayed the lowest cover in hermaphrodite broadcaster branching hard corals such as Acroporidae (Fig. 4b).

**Table 1:** Community-weighted-mean values (CWM) for the different traits and benthic communities studied.

Regime	Colonial	Form	Flexibility	FS	Photosynthetic symbionts	RS	Sexual system	Skeleton	CMW	GR	Colony size	Longevity
Ba1	Y	Mass	3	Sel	3	Br	H	N	1	3	1	2
Ba2	Y	Bra	1	Sel	3	Bc	G	Y	1	3	3	4
Ba3	Y	Encr	1	FF	2	Al	H	N	1	3	2	1
BuD	Y	Mass	1	FF	2	Al	H	N	1	1	3	4
BuS	Y	Bra	1	Sel	3	Bc	H	Y	1	1	3	4

Body form categories: *Mass.* massive, *Bra.* branching, *Encr.* encrusting. FS categories: *Sel.* selective, *FF* filter-feeder. RS categories: *Br* brooder, *Bc.* broadcasters, *Al.* alternate. *FS* feeding strategy, *RS* reproductive strategy, *CMW* corallite maximum width, *GR* growth rate.

BuD presented the highest taxonomic and FE richness (95%), however it presented a smaller functional richness (90%) than BuS (95%) (Fig. 4b,c). BuD sites were dominated by massive, long-lived, slow-growing filter-feeding species, such as barrel and massive sponges; whilst BuS was characterised by branching, calcified, long-lived, broadcaster species such as Acroporidae corals (Table 1, Fig. 4b). Both BuD and BuS contained most of the FEs contributing to reef accretion and reef structural complexity (Fig. 4b).

Out of the 64 FEs and 99 high-resolution taxonomic categories, only 29 FEs and 28 taxonomic categories were found in all sites (Supplementary Figure 2). BuD was the site with the highest number of unique FEs (9) and unique taxonomic categories (11), but none of the Bangka sites presented any unique benthic FEs or taxonomic categories (Supplementary Figure 2). Bunaken communities (BuD and BuS) presented 2 FEs that were absent from the sites studied at Bangka island. Deep sites (BuS, Ba1 and Ba3) presented two FEs that were absent in the shallow sites, whereas the shallow communities (BuS and Ba2) had one unique FE, the blue coral *H. coerulea* (Supplementary Figure 2).

### Functional diversity analysis of fish communities

**Figure 5:**
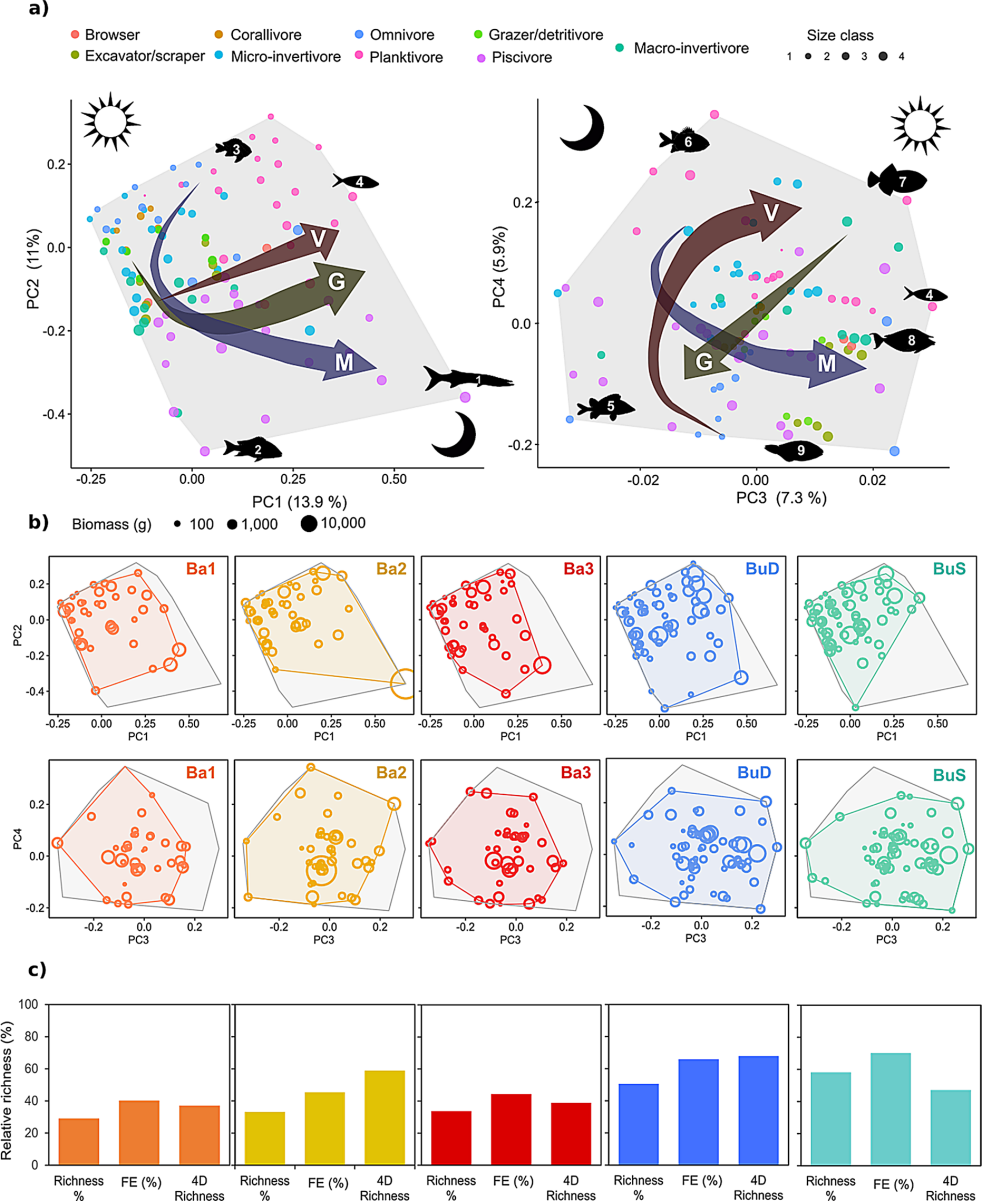
Fish functional diversity of the different communities identified. (**a**) Distribution of functional entities (FEs) in the global fish functional space, built using four PCoA axis (PC1 and PC2 left, PC3 and PC4 right) using six functional traits: body size, diet, period of activity, vertical position (V), gregariousness (G) and mobility (M). The numbers indicate the following functional entities: 1: *Sphyraena quenie* (Sphyraenidae), 2: big snappers (e.g., *Macolor macularis,* Lutjanidae), 3: damselfishes, 4: pelagic planktivores such as fusiliers (Caesionidae), 5: squirrelfishes (*Sargocentron* spp., Holocentridae), 6: soldierfishes (*Myripristis* spp., Holocentridae), 7: *Melichthys vidua*, 8: unicornfishes (*Naso* spp., Acanthuridae). (**b**) Functional spaces of each of the communities’ analysed (coloured convex hull) superposed to the global functional space (grey). The bubble sizes represent the FEs mean biomass at each of the regimes. (**c**) Functional diversity indices for each of the communities: relative taxonomic richness (Richness %), relative FE richness (FE %) and relative functional richness as % of filled global functional space (4D richness).

We identified 172 fish species that were classified into 97 FEs (Supplementary Table 3), for which their functional niche was displayed using a functional space built on four PCoA axis. Generally, gregariousness and vertical position changed along the first axis of the functional space (PC1) (Fig. 5a). The second axis (PC2) was characterised by differences between nocturnal and diurnal species, fish size and diet, showing a clear separation between planktivorous (e.g., damselfishes, fusiliers) and piscivorous fish (e.g., snappers, barracudas). Fish mobility was captured by both PC1 and PC2. PC3 showed a clear separation between nocturnal (left) and diurnal (right) species, and also captured fish mobility, with highly mobile fish species such as fusiliers or surgeonfishes at the right extreme of the functional space. Fish gregariousness also changed along PC4, but the pattern was not as clear as with PC1. Vertical position also changed with PC4, with highly substrate associated species such as parrotfishes or squirrelfishes at the bottom of the functional space (Fig. 5a).

All Bangka sites displayed the lowest taxonomic and FE richness (S_richness_ = 29–33%, FE_richness_ = 40–45%). Ba1 and Ba3 also presented the lowest functional richness, filling only 37 and 39% of the functional space respectively. Ba2, however, exhibited the second highest functional richness, filling 59% of the functional space (Fig. 5b,c), which was related to the presence of few highly original FEs such as big highly mobile predators (i.e., barracudas, PC1) and the nocturnal, gregarious, highly-site attached omnivorous sweeper (*Pempheris oualensis*, PC3–PC4), which were uniquely found in Ba2 (Fig. 5b). Overall, all Bangka sites were characterised by the lack of browsers; although this trophic group was only represented by two FEs consisting of three Acanthuridae species (Data S2). Ba1 and Ba3 also displayed low functional diversity and biomass of grazers/detritivores. Ba1 and Ba2 lacked the presence of most big, high-trophic chain fish (i.e., piscivorous, macro-invertivore and omnivorous), excepting the barracudas in Ba2 (Fig. 5b).

Bunaken sites harboured the highest number of fish species and FEs, which were characterised by middle-size, diurnal planktonic species (Table 2, Fig. 5b,c). BuD, which harboured 50% of all species and 66% of all FEs, displayed the highest functional richness (68%). BuS, which hosted the largest taxonomic richness (58%) and FE richness (70%) only filled 47% of the functional space, which was related to the absence of nocturnal piscivorous species such as snappers or barracudas (Fig. 5b,c).

**Table 2:** Community-weighted-mean values (CWM) for the different traits and fish communities studied.

Regime	Size	Mobility	Activity	Gegariousness	Vertical position	Diet
Ba1	3	3	0	3	1	Piscivore
Ba2	6	4	1	4	3	Piscivore
Ba3	4	3	0	3	1	Piscivore
BuD	3	3	0	4	2	Planktivore
BuS	3	3	0	1	2	Planktivore

Out of the 97 FEs and 172 species identified in all communities, only 19 FEs and 13 species were present in all sites (Supplementary Figure 2). None of the piscivorous FEs was shared between all sites, but, all sites presented unique piscivorous FEs. BuD was the community with the highest number of unique FEs (11), followed by BuS (9 FEs) which was however the site with the most unique number of species (26). The Bangka sites all displayed lower number of unique FEs and species ranging from 3–4 unique FEs and 9–12 unique species (Supplementary Figure 2). Bunaken communities had 9 FEs that were absent in the communities studied in Bangka island, which were mostly medium to highly mobile FEs such as fusiliers (Caesionidae) and large Acanthuridae (Fig. 5, Supplementary Figure 2). Shallow sites (BuS and Ba2) presented four unique FEs that were absent from deep sites (BuS, Ba1 and Ba3) (Supplementary Figure 2).

## Discussion

As coral reef communities change and reorganise in response to increasing anthropogenic and climate disturbances, approaches that detect new species configurations and their contribution to key ecosystem processes are required^42,43^. Here, we selected reefs with different natural characteristics and exposed to anthropogenic factors, which we hypothesised would display different fish and/or benthic regimes, to compare the use of different biodiversity approaches. We show that the use of major categories (family level or above) in studying coral reef communities fails to identify distinct regimes. We also implement the use of a trait-based approach to study coral reef fish and benthic communities, and show its relevance in the study and detection of different communities.

The spatio-temporal study of coral reefs is key to predicting their trajectories and recovery potential after disturbances^17^. Within this context, global citizen science programs are vital for the temporal study of coral reefs, and contribute to community capacity building and education^23^. Such programs, however, rely often on the study of coral communities using major categories (at family level for fishes and at class level or higher for benthic organisms), which as we show here might mask the presence of distinct assemblages. In fact, our results showed that by using major categories we not only detected fewer regimes, but they were also less consistent across the different organisms studied (corals, benthos, fish), possibly suggesting lower ecological relevance or accuracy of the regimes identified. For example, the study of major benthic categories mixed Ba1 (dominated by fast-growing xeniids), with Ba2 (dominated by the hydrocoral *H. coerulea*) and BuS (dominated by branching fast growing scleractinian corals such as Acroporidae), which were clearly separated by using functional entities and high resolution taxonomic categories. Communities dominated by branching scleractinian corals (such as in BuS), are generally examples of healthy, highly complex coral reefs^7,26^, whilst communities dominated by xeniids (such as Ba1) tend to display much lower structural complexity and might be characteristic of degraded habitats^44^. Given that current global change scenario is resulting in unprecedented ecosystem degradation, temporal monitoring of ecosystems and the detection of community changes is of foremost importance^21,45,46^. However, the monitoring approaches might need to be readjusted or extended in order to provide higher taxonomic resolution surveys that capture the different emerging species configurations as previously suggested by Jouffray et al. (2015), Lam et al. (2017), and Donovan et al. (2018)^21,45,46^. Furthermore, our results also agree with Smith et al. (2016), which highlighted the importance of including non-coral benthic organisms in monitoring^47^.

Changes in coral reef communities and especially the decline of key reef building species contribute to the long-term functional erosion of coral reefs that could result in the loss of associated ecosystem services^9,16^. For example, decreases in structural complexity and the associated loss of habitat structure have been associated with a decline in fish biomass and therefore fisheries^16^. However, as shown recently in some Caribbean reefs, not all communities with low-coral cover might display compromised ecosystem functioning^42^, highlighting the need to understand the composition but also functioning of different coral reef communities. The use of trait-based approaches to gain insights into the role of biodiversity in ecosystem functioning has been successfully implemented to study and detect changes in fish and scleractinian coral communities^26,29^, but to date such approaches have not yet been implemented to study coral reef benthic changes beyond scleractinian corals. Identification of coral reef benthic organisms to species or even genus level is extremely challenging, especially from visual census or imagery^48^, which probably has restrained researchers from applying trait-based approaches to whole coral reef benthic communities. Many benthic organisms are highly understudied^49^, whilst others such as sponges or soft corals require advanced genetic tools or microscopic examination for their taxonomic classification^50–52^. Here, we show that even if visual identification of many coral reef benthic organisms to species level remains impossible, the classification of organisms at lower levels, which for some organisms may just be at the morphological level (i.e., sponges)^53^, still yields high quality data on which trait-based approaches can be applied. Within our trait-based approach, we used ordered categorical traits instead of continuous traits (e.g., growth ranges and broad lifespan ranges instead of specific values) in order to consider the inherent trait variability from categories that contain several species (e.g., *Acropora* spp.). The use of such an approach allowed us not only to delineate different community regimes that matched the ones identified using fish communities (at species level), but also to obtain insights into some of the functions that might be compromised in the different community regimes.

Our results show that coral reef assemblages around Bangka and Bunaken islands are highly heterogeneous as previously highlighted by Ponti et al*.*^35^. More importantly, we observed that out of five different community regimes detected, only two were dominated by reef-building species, one of which was dominated by the blue coral *H. coerulea*. Dominance of the blue coral in other Indo-Pacific reefs has been previously reported and has been attributed to high growth, high thermal tolerance and its capacity of inhibiting scleractinian coral larval recruitment^54,55^. Under the present scenario of climate change, communities dominated by *H. coerulea* might become increasingly common, but to date, there is little information if *H. coerulea* dominated communities might sustain similar ecosystem functions as scleractinian dominated reefs^55^. Here, we show that the community dominated by *H. coerulea* (Ba2) presented comparable benthic and fish functional diversity to the scleractinian-dominated regime (BuS). However, we would like to note that our surveys were one-time diurnal surveys and therefore a temporal data series is required to analyse the community temporal trajectories and temporal changes in functional diversity within sites. Such data would provide further insights on whether some of the observed regimes (i.e., dominated by *Heliopora*) have long-term negative functional impacts, or if they are just new regimes that can sustain key ecosystem functions (e.g., reef accretion, structural complexity).

The approach used also allowed us to identify two regimes that were dominated by potentially proliferating non-calcifying invertebrates displaying fast-growths and short lifespans. The proliferation of invertebrates able to overgrow live corals such as ascidians, sponges, or some soft corals such as the opportunistic xeniids has been previously linked to the degradation of environmental conditions^56–59^. The dominance of benthic habitats by the soft coral *Xenia* spp. has been previously observed at different Indonesian reefs affected by blast fishing, including some reefs in Bunaken, Komodo and Wakatobi national parks^44,60^. Proliferation of colonial ascidians has also been reported on several reefs after increases in nutrient availability and overfishing^58,61^. Increases in these organisms can have severe effects on reef health and functioning by altering reef replenishment, geomorphology and trophic structure^58,62,63^. Here, we observed that the two communities dominated by potentially proliferating organisms displayed simultaneously the lowest fish and benthic functional diversities, including the lowest diversity and cover of reef-building functional entities, such as branching corals. Many ascidians, soft corals and sponges possess varied chemical defences, that not only offer them spatial competitive advantages over corals, but that can also contribute to the inhibition of coral settlement, further contributing to coral loss^64–66^. Proliferation of such organisms therefore poses a serious threat to the stability of coral reef ecosystems, however more studies are needed to further explore their implications on ecosystem functioning and their temporal persistence.

Sponges are the second most important invertebrate group (after corals) in determining substrate composition and nutrient cycles in coral reefs^67^. Increasing evidence suggests that sponges might be increasing in abundance as consequence of climate change and anthropogenic stressors such as eutrophication, overfishing or sedimentation^13,57,68^. Whereas the temporal persistence of sponge reefs and their functioning is still unclear and the subject of much current discussion^69,70^, a misleading constraint in hitherto existing studies is potentially the consideration of sponge reefs as a homogeneous entity. In fact, sponge species display marked divergence in their morphotypes and life histories, with some encrusting sponges such as *Terpios hoshinota* displaying turnover rates of months, whereas barrel sponges live hundreds to thousands of years^71–73^. Therefore, a sponge reef dominated by fast-growing short-lived sponges such as encrusting or bioeroding sponges might function completely different than a sponge reef composed mainly of slow-growing long-lived sponges, highlighting the need for the detailed study of such habitats and organisms’ functions. Here, all the sites surveyed at 10 m in the island of Bunaken were dominated by sponge communities. Interestingly, when the composition was studied using the taxonomic categories, we identified encrusting sponges as the major taxonomic benthic group in this regime. However, when we explored the functional diversity and identified the most often encountered functional traits (CWM), we were able to observe that this regime was in fact characterised by long-lived, slow-growing, massive, filter-feeder organisms, which included both massive and barrel sponges. These results demonstrate the importance of studying communities at the functional level, since it enables a much more comprehensive understanding of the community and their ecosystem functioning (e.g., by considering similar traits from different taxonomic groups) than taxonomic categories. We also observed that the sponge-reefs observed in Bunaken displayed the highest fish functional diversity, the second highest benthic functional diversity, and contained most of the benthic functional entities contributing to reef building and accretion. Although, as mentioned earlier, more surveys both in space and time are required to draw solid conclusions on the trajectories of coral reef communities around Bunaken and Bangka islands, our results suggest that the sponge-reefs identified might not be related to changing environmental conditions, but rather to other inherent reef characteristics such as topography (i.e., all the sponge-reef sites were reef walls).

In summary, by using a case study on coral reefs at the epicentre of tropical marine biodiversity we provide new evidence on (1) the importance of using high resolution taxonomic data for the detection of community regimes, (2) the usefulness of using trait-based approaches to explore and identify different community regimes and their contribution to key ecosystem processes and (3) the necessity of considering all benthic organisms to detect species configurations that are not dominated by scleractinian corals.

## Methods

### Coral reef surveys

Benthic and fish surveys were conducted at nine different sites at Bunaken and Bangka islands (North Sulawesi, Indonesia) between February and March 2020 (Fig. 1). The sites were chosen due to their high heterogeneity (i.e., reef topography, exposure to anthropogenic impacts) in order to reflect different community regimes around these two islands (Supplementary Table 1). Six sites were chosen within the Bunaken National Park (around Bunaken island, 8 km^2^, ≈ 7500 habitants), which regulates human activities such as fishing (only traditional fishing and harvesting is allowed)^74^. Three sites where monitored in the neighbour island of Bangka (48 km^2^), which has a resident population of 2500 habitants (as of 2013^35^). At the time of the surveys, none of the Bangka sites were a Marine Protected Area. Two of the Bangka sites (Ba1 and Ba2) were located in front of a resort, where non-destructive fishing practices such as hook and line and spear fishing take place. Ba3 site (Sipi) has been exposed to higher fishing pressure, including destructive fishing practices and is also located in front of an iron ore mine that was established between 2013–2015 (Supplementary Table 2^35^). Three 20-m transects (separated by 5 m) were placed at each site parallel to the coast at either 3 or 10 m deep (Fig. 1). The benthos and fish communities were characterised on each transect using three different levels of ecological information: major categories, which have been used in citizen science programs such as Reef Check, categories at the highest taxonomic resolution possible (e.g., genus or species) and functional entities (Supplementary methods).

To assess the benthic composition, we photographed the benthos every 0.5 m from a distance of 0.5 m above reef bottom using a camera (Olympus TG-5) mounted on a squared metal frame covering a surface area of 0.25 m^2^. All photographs were analysed using the software Coral Point Count Excel extension^75^ to determine the percentage cover of different sessile organisms (Supplementary Data 1). Thirty random points were assigned to each photograph and the organisms under these points were identified to the lowest taxonomic level possible (n = 1200 points per transect, 99 benthic categories identified, Supplementary Table 2). These taxonomic categories were classified into seven major categories: hard coral, soft coral, sponge, coralline algae, macroalgae, fleshy algae and other living organisms. Scleractinian corals were also classified into 11 sub-categories according to their morphology: branching, caespitose, columnar, corymbose, digitate, foliose, massive, submassive, solitary and tabular, which were used as major categories in the analyses of only scleractinian coral communities (Supplementary Table 1, Supplementary methods).

For the fish survey, 3-min videos were taken swimming along the transects at a constant speed. A first video was filmed while laying the transect and was used to collect data on bigger fish species that might be scared away by the divers. A second fish video was filmed five minutes after laying the transect and was used to count the resident fish species. All fishes (> 5 cm) within 3 m on each side of the transect and 3 m above were counted, their family and species identified and their approximate length (± 3 cm) recorded. Larger and rarer fish (e.g., unicornfishes, parrotfishes, big groupers, pelagic fish such as barracudas and trevallies) were counted when observed within 5 m from the transect. Fish biomass was calculated following Froese (2006)^76^ using the equation W = a × L^b^, where W is the weight of the fish in grams, L is the total length (LT) in cm and a and b are species-specific constants obtained from FishBase^77^ (Supplementary Data 2). Fish families were used as major categories, whereas fish species were used for the high taxonomic resolution analysis (Supplementary Table 3).

### Benthic and fish traits

The functional ecology of benthos categories was characterised using 12 traits: colony formation, growth form, maximum colony size, longevity, growth rate, body flexibility, skeleton presence, reproductive strategy, sexual system, feeding strategy, presence of photosynthetic symbionts and corallite maximum width (only for scleractinian corals). The chosen functional traits focus on key ecosystem processes that affect the organisms’ population dynamics, coral reef accretion and nutrient cycling and resources. Since category identification was performed at genus or higher taxonomic ranks, we often used ordered categories to classify the quantitative traits (Supplementary Methods). Most of the functional traits from scleractinian corals were extracted from the Coral Trait Database (https://coraltraits.org/)^30^, whereas scientific references and monographs were used to extract information on the other organisms traits (Supplementary Table 2).

The functional ecology of fish species was characterised using six traits (body size, diet, period of activity, vertical position, gregariousness and mobility) (Supplementary Methods, Supplementary Table 3). The chosen traits describe the main facets of fish ecology and are relevant in critical ecosystem processes such as nutrient cycling and food web regulation^29^. Fish trait data was collected from the FishBase database (http://www.fishbase.org) and from published articles studying coral reef fish functions^29,78,79^. Unique combinations of traits were defined as functional entities (FEs).

### Multivariate community analysis

All numerical analysis were performed on R version 3.6.1^80^. Benthic cover and fish biomass data were transformed using the Hellinger transformation (function “decostand” from the vegan R package^81^) prior to the multivariate analyses. Non-metric multidimensional scaling (NMDS, metaMDS function from the vegan package) analyses based on Euclidean distance were used to visualize the differences in benthos (scleractinian corals and all benthic animals) and fish communities between the different sites. The three levels of ecological information (major categories, categories defined at the lowest taxonomic level possible and functional entities) were used to investigate how they affect community pattern detection. A hierarchical cluster analysis (function hclust in R) using Euclidian distance was then used to identify clusters of similar sites (i.e., regimes). The “Average” algorithm was chosen after analysis of the cophenetic correlation coefficient (Pearson correlation between the cophenetic distances calculated on cluster branches and the benthos/fish community dissimilarity matrix). The Kelley-Gardner-Sutcliffe (KGS) penalty function (maptree R package) was used to prune the dendrogram and obtain the optimal number of clusters. This function maximises differences between groups and cohesiveness within groups (using the species pairwise distance matrice)^82^. The different clusters identified were considered hereafter as compositionally different community regimes and were used for the subsequent description of the communities using high taxonomic resolution data and functional analysis.

### Functional space and functional indices

The functional richness was calculated as the volume within the multidimensional functional space enclosing all the FE in a specific community, where each species is placed according to their functional niche^83,84^. First, a species dissimilarity matrix was built using the Gower’s distance^85^. This distance was first implemented in functional diversity analyses by Pavoine et al. (2009) due to its capacity of dealing with different types of traits (continuous, ordinal and categorical), its efficiency in dealing with missing data as well as allowing inclusion of variable weights^86^. Nowadays, it is one of the most commonly used distances in functional diversity analysis, and is specially recommended to detect changes in marked different communities^87^. Then, a Principal Coordinates Analysis (PCoA) was performed using the previous dissimilarity matrix. In order to select the number of PCoA axis that would result in the best functional space, which needs to be congruent with the initial functional distance, we computed the mean squared deviations (mSD) of functional spaces with multiple axis (up to 10), in which lower mSD represents a higher quality of the functional space^84^. After examination of mSDs, we selected four axis to build both of our functional spaces (for benthos and fish), since adding a fifth axis only weakly increased the quality of the functional spaces (Supplementary Figure 3, Supplementary Figure 4). The functional space and the mSD values were computed using the R function quality_funct_space, developed by Maire et al*.*^84^.

The number of FE (FErichness) was calculated to explore the functional diversity. We also computed the community-weighted means of trait values (CMW) using the dbFD function from the FD R package^88^. The CMW provides information on functional composition by identifying the most common value traits in a specific community.

## Supplementary Information

Supplementary Data S1.

Supplementary Data S2.

Supplementary Information.

## Data Availability

All data generated or analysed during this study are included in this published article (and its Supplementary Information files).
